# Sequential crystallization pathways in apatite–wollastonite glass-ceramics *via* spray pyrolysis

**DOI:** 10.1039/d5ra08885b

**Published:** 2026-03-18

**Authors:** Andualem Belachew Workie, Mannie Belay Taye

**Affiliations:** a Bahir Dar Institute of Technology H9XW+4MP Bahir Dar Ethiopia andualembelachew2@gmail.com; b Injibara University Ethiopia

## Abstract

The clinical performance of next generation bioceramics requires precise control over their multi-phase composition, a goal often unachievable with conventional synthesis routes that lead to stochastic crystallization. Here, we establish a deterministic processing-structure paradigm for apatite–wollastonite glass-ceramics (AWGCs) by using a scalable spray pyrolysis technique to create atomically homogeneous amorphous precursors. By systematically controlling the sintering temperature (700–1100 °C), we decouple the kinetic and thermodynamic drivers of phase formation. High-resolution XRD with Rietveld analysis reveals a predictable, three-stage evolution; kinetically driven hydroxylapatite nucleation (<800 °C), followed by a thermodynamic crossover to a whitlockite-dominant regime (900–1000 °C), and finally the diffusion-controlled growth of mechanically robust wollastonite (>1000 °C). This work presents the first quantitative, predictive map for this system, enabling the rational design of AWGCs with bespoke phase assemblages for targeted biomedical applications, from bioactive coatings to load-bearing implants.

## Abstract 2

A predictive phase-temperature map reveals sequential crystallization of apatite–wollastonite glass-ceramics, advancing rational design of bioceramics.

## Graphical abstract

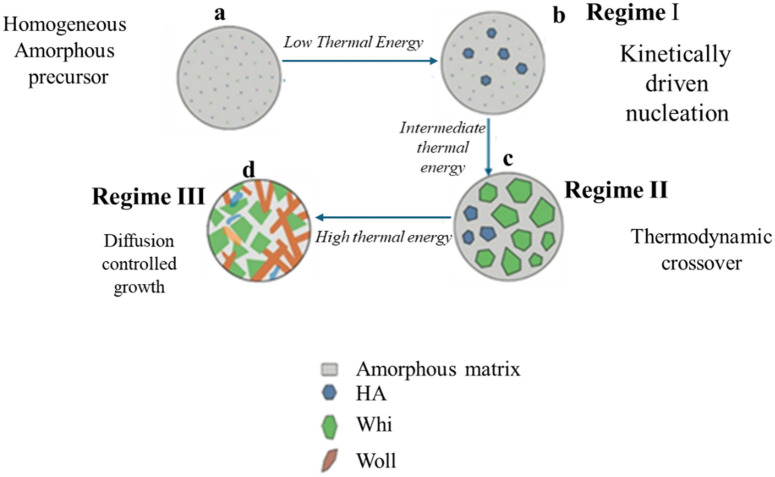

## 1. Introduction

A basic materials trilemma confronts the design of advanced orthopedic and dental implants: striking a balance between the mechanical toughness necessary for load-bearing applications, the controlled resorbability required to promote natural tissue regeneration, and the bioactivity necessary for osseointegration.^1–3^ By integrating bioactive phases like hydroxylapatite (HA), a resorbable phase like whitlockite (Whi), and a mechanically reinforcing phase like wollastonite (Wol), apatite–wollastonite glass-ceramics (AWGCs) are a superior class of biomaterials created to meet this difficulty.^4–7^ AWGCs implant's final clinical success is determined by the cooperative interaction of its phase assemblage rather than by a single element. Therefore, the crucial barrier preventing these materials from being translated from the lab into custom therapeutic treatments is the capacity to accurately and consistently regulate this multiphase architecture.

This degree of control is not achievable with current synthesis techniques, such as conventional melt-quenching and sol–gel approaches. Melt-quenching requires a lot of energy and is prone to chemical segregation, which produces heterogeneous nucleation sites with poor phase selectivity and wide crystallization windows.^8^ Although sol–gel approaches provide more homogeneity, they need intricate, multi-step processing to eliminate organic residues, which may unintentionally alter crystallization routes.^8,9^ Because of the intrinsic stochasticity of nucleation in these techniques, a real priori design of the final phase composition is extremely difficult, forcing manufacture to rely on empirical, trial-and-error procedures.

By using a continuous, single-step pyrolysis approach to create AWGC precursors, our study gets beyond these basic restrictions. By enabling remarkable chemical homogeneity at the nanoscale within every precursor particle, this aerosol-based technique essentially creates a blank slate for crystallization that is devoid of the haphazard nucleation sites that beset other approaches.^10–12^ We speculate that during thermal processing, the deconvolution of thermodynamic and kinetic driving forces is made possible by this exceptional precursor homogeneity. This makes it possible to create a very predictable and kinetically controlled phase development pathway in which different crystalline phases appear in discrete, well defined temperature ranges according to their own growth and nucleation barriers.

Building on this foundation, our study tackles a key challenge in bioceramic engineering: establishing a reliable, mechanistic framework for designing multiphase AWGCs. By leveraging spray pyrolysis to ensure both scalability and nanoscale homogeneity, we bridge the gap between empirical fabrication and rational design. This enables us to provide a clear and practical roadmap for balancing and optimizing the essential properties of bioactivity, resorbability, and mechanical strength in advanced bioceramics.

## 2. Materials and methods

### 2.1 Precursor synthesis *via* spray pyrolysis

A multicomponent aqueous precursor solution based on the MgO–CaO–SiO_2_–P_2_O_5_–CaF_2_ system was formulated as described in our previous work.^13^ The solution was atomized into a fine aerosol using an ultrasonic nebulizer (1.65 MHz) and passed through a tube furnace for in-flight decomposition and calcination. This continuous process yielded a fine, chemically homogeneous, and amorphous-dominant powder.

### 2.2 Controlled thermal processing and sintering

The as-synthesized powder was uniaxially pressed into cylindrical pellets (Ø 13 mm, 60 MPa). The pellets underwent a controlled sintering protocol in an air-filled furnace. A heating rate of 5 °C min^−1^ was used to reach discrete target temperatures of 700, 800, 900, 1000, and 1100 °C. Samples were held at the peak temperature for a 4 hours soaking period to approach phase equilibrium, followed by furnace cooling. The sintering temperature was the principal experimental variable.

### 2.3 Crystallographic and microstructural analysis

Phase identification and quantification were performed using high-resolution X-ray Diffraction (XRD) on crushed pellets (Bruker D2 Phaser, Cu Kα radiation, 2*θ* range 20° to 80°). The diffraction patterns were analyzed to identify the primary crystalline phases, and major peaks were identified as hydroxylapatite, whitlockite, and wollastonite. Quantitative phase analysis was conducted using the Rietveld refinement method with TOPAS v6 software. The model incorporated instrumental parameters, a Chebyshev polynomial background, and refined scale factors, unit cell parameters, and crystallite size/micro strain for each phase using a double-Voigt approach. Due to the complex, multiphase nature of the glass-ceramic, atomic positions and bond lengths were constrained to their standard crystallographic models to ensure refinement stability and physical realism. High-quality fits were ensured by targeting weighted-profile *R*-factors (*R*_wp_ < 10%) and goodness-of-fit (*χ*^2^) values near unity. Bulk density was measured *via* the Archimedes' method, with three measurements averaged per sample. Microstructure of fractured pellet surfaces was examined using field emission scanning electron microscopy (FESEM).

## 3. Results

### 3.1 Characterization of the as-synthesized precursor

The XRD pattern of the as-pyrolyzed powder confirmed a primarily amorphous structure with only trace evidence of incipient apatite nanocrystals, consistent with a highly reactive, glassy starting material. Complementary microstructural analysis *via* FESEM revealed a uniform collection of spherical particles, characteristic of the spray pyrolysis process. Quantitative image analysis (*n* = 500 particles) determined a mean particle diameter of 0.92 ± 0.44 µm. The particle size distribution histogram is provided in the supplementary information (Fig. S1). Furthermore, the chemical homogeneity of this precursor, a cornerstone of this study's deterministic approach, was verified by EDS. The analysis confirmed that the experimental elemental composition closely matched the theoretical stoichiometry derived from the precursor solution, ensuring that phase evolution was not biased by compositional segregation. A representative EDS spectrum and the quantitative comparison are provided in SI (Fig. S2 and Table S2).

### 3.2 Temperature-dependent phase evolution

**Fig. 1:**
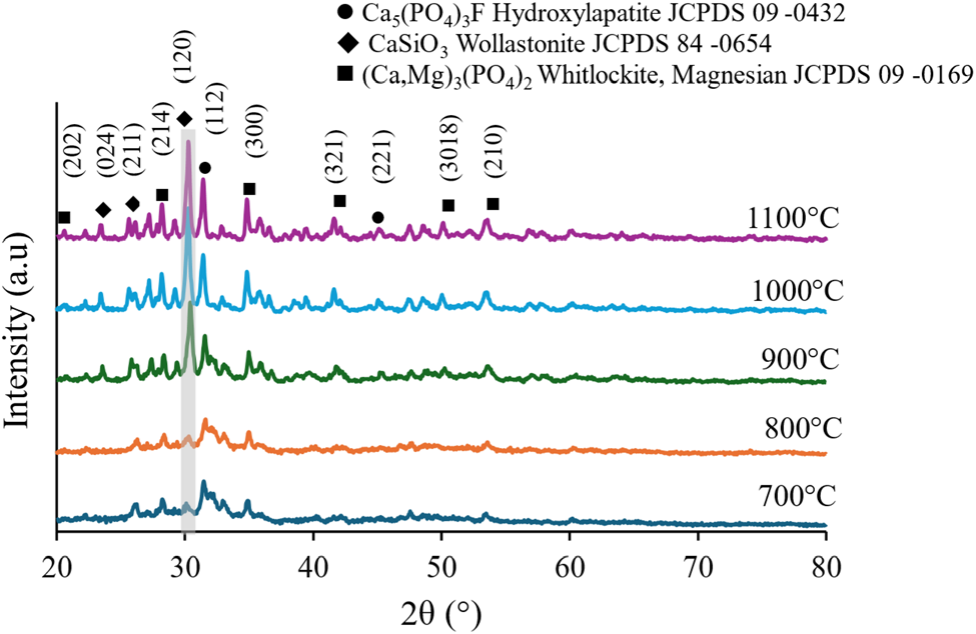
High-resolution X-ray diffraction (XRD) patterns of AWGCs sintered at temperatures from 700 to 1100 °C, revealing a clear temperature-dependent phase evolution.

Fig. 1 High-resolution XRD patterns of AWGC samples sintered at temperatures from 700 °C to 1100 °C for four hours. The patterns show a clear temperature-dependent phase evolution from a largely amorphous state at 700 °C to a highly crystalline, multiphase ceramic at 900 °C and above. The primary crystalline phases identified are hydroxylapatite (HA, JCPDS 15-0876), whitlockite ((Ca,Mg)_3_(PO_4_)_2_, JCPDS 13-0404), and wollastonite (Wol, CaSiO_3_, JCPDS 76-0186). Key diffraction peaks are labeled with their corresponding (*hkl*) Miller indices. The highlighted region (gray bar) around 30.5° 2*θ* emphasizes the pronounced crystallization and sharpening of the primary wollastonite (120) and HA (112) peaks as the sintering temperature increases.

The temperature-dependent phase evolution of the SP AWGCs was characterized by high-resolution XRD. At 700 °C, the pattern exhibits broad humps characteristic of a primarily amorphous material, with only incipient, poorly defined peaks. As the temperature increases to 800 °C, the initial crystallization of HA is observed, evidenced by the appearance of the (112) peak.

A dramatic transformation occurs between 800 °C and 900 °C, where the material becomes highly crystalline. This transition is dominated by the emergence of the Whitlockite phase, identified by its characteristic peaks. Concurrently, the most intense peak in the pattern, located in the highlighted region at ∼30.5° 2*θ* and 31.5° corresponding to the wollastonite (120) and HA (112) planes, sharpens significantly. From 900 to 1100 °C, the relative intensity of the whitlockite and HA peaks, such as the one at 41.5° 2*θ* (321) and 44.5° 2*θ* (221), continues to increase, indicating the growth of this mechanically robust phase at the highest sintering temperatures.

### 3.3 The quantitative phase-temperature map

**Fig. 2:**
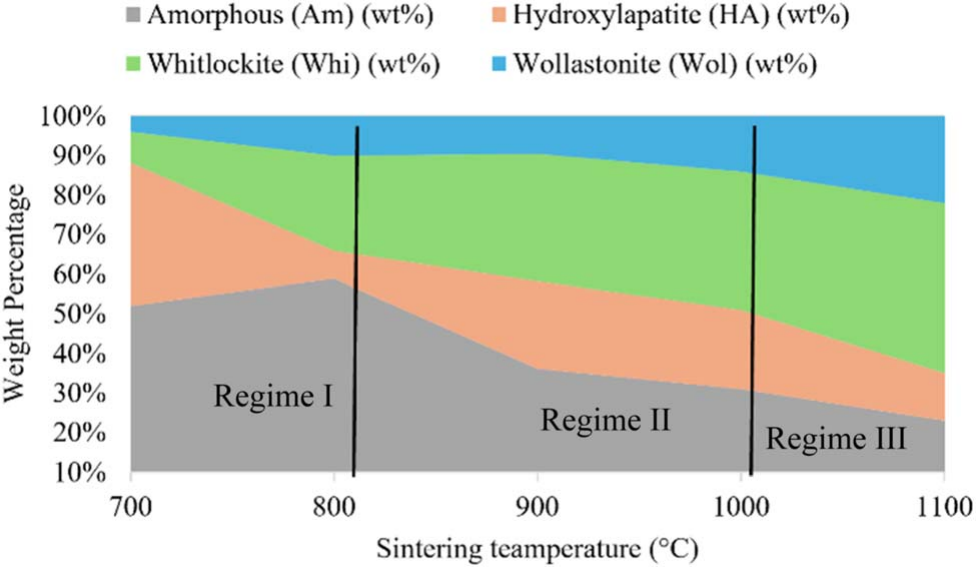
The quantitative phase-temperature map for spray-pyrolyzed AWGCs the competitive and sequential phase transformations that define the material's final composition. Regime I (700–800 °C): the material is dominated by the amorphous matrix, with initial kinetic nucleation of HA. Regime II (800–1000 °C): a thermodynamic crossover leads to the rapid crystallization of Whi, which becomes the dominant crystalline phase at the expense of the amorphous content. Regime III (1000–1100 °C): diffusion-controlled growth of mechanically reinforcing Wol occurs, marking the final stage of crystallization toward a stable, high-temperature ceramic.

Rietveld refinement of the XRD data enabled detailed quantification of each phase, resulting in a comprehensive phase, temperature map (Fig. 2).

In regime I (700–800 °C), the material retained a high amorphous content, decreasing only slightly from over 55% to about 50%. During this stage, HA began to crystallize, its fraction increasing to roughly 38%, while Whi and Wol remained virtually absent. Transitioning to regime II (800–1000 °C), the system experienced pronounced crystallization. The amorphous phase dropped rapidly below 25%, while the Whi phase surged to a peak of approximately 28 wt% at 900 °C, emerging as a major component alongside HA. Finally, in regime III (1000–1100 °C), the formation of the Wol phase accelerated. Previously present in only trace amounts, Wol grew to about 22 wt% by 1100 °C. This development occurred at the expense of both HA and Whi, whose phase fractions diminished as sintering progressed to the highest temperatures.

### 3.4 Microstructural evolution and densification

**Fig. 3:**
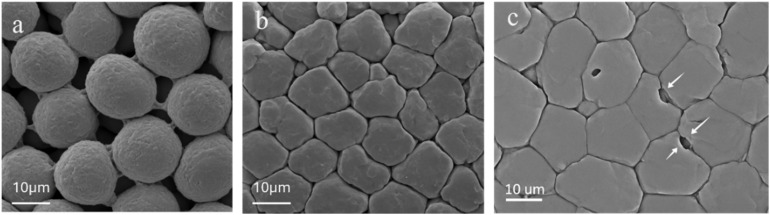
Microstructural evolution of sintered AWGCs, which directly correlates with the phase transformations (a) at the regime I/II boundary (800 °C), initial particle necking and a porous, nanocrystalline structure are evident. (b) By the regime II/III boundary (1000 °C), pronounced crystallization has led to a dense, consolidated grain structure with minimal interparticle porosity. (c) In regime III (1100 °C), the growth of wollastonite is accompanied by significant grain coarsening and the appearance of entrapped, intragranular pores (indicated by arrows), which slightly reduces the bulk density.

The phase transformations directly correlated with microstructural changes observed *via* FESEM (Fig. 3). At the regime I/II boundary (800 °C), initial particle necking was evident (Fig. 3a). By the regime II/III boundary (1000 °C), substantial crystallization led to a dense, consolidated grain structure with minimal interparticle porosity (Fig. 3b). At 1100 °C, significant grain coarsening was observed, along with the appearance of entrapped, intragranular pores (Fig. 3c).

**Fig. 4:**
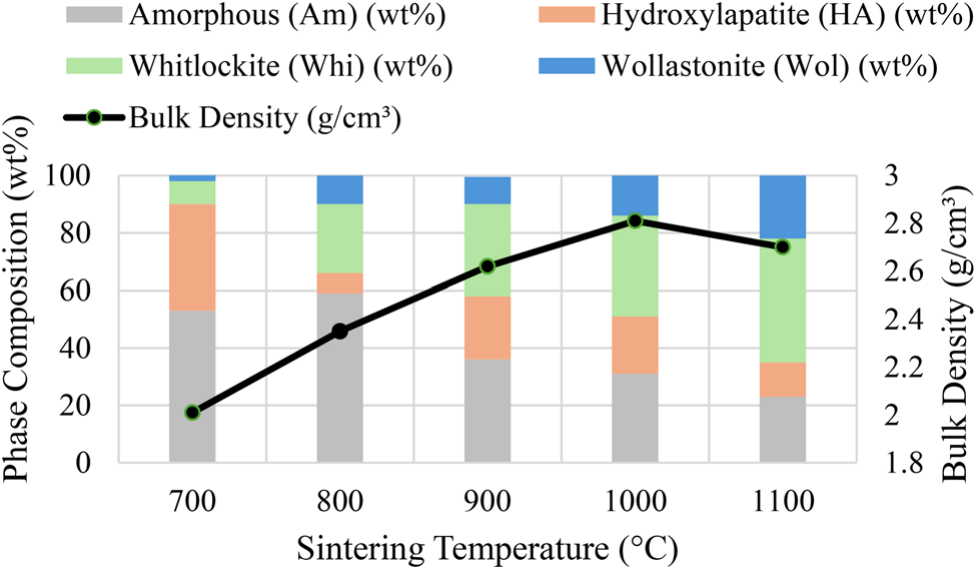
Correlation between crystallographic phase evolution and macroscopic densification in sintered AWGCs. The stacked columns represent the quantitative phase composition (wt%) at each sintering temperature. The overlaid line chart plots the corresponding bulk density (g cm^−3^). This visualization clearly demonstrates that the densification of the material is driven by crystallization-induced pore removal, with peak density (2.81 g cm^−3^) achieved at 1000 °C, corresponding to the point of maximum crystallinity. The slight density decreases at 1100 °C is attributed to grain coarsening that occurs during wollastonite formation.

This microstructural evolution drove macroscopic densification, as plotted in Fig. 4. Bulk density increased steadily from 2.25 g cm^−3^ at 800 °C to a maximum of 2.81 g cm^−3^ at 1000 °C. Beyond this point, the density slightly decreased to 2.78 g cm^−3^ at 1100 °C, despite the formation of the intrinsically dense Wol phase.

## 4. Discussion

The results present a clear and deterministic relationship between processing temperature and phase assemblage in AWGCs synthesized from a homogeneous spray-pyrolyzed precursor. This predictability stands in stark contrast to the stochastic crystallization common in conventional synthesis routes and provides the foundation for a rational design paradigm. The phase-temperature map (Fig. 2) is not merely a description of outcomes; it is a processing blueprint derived from the underlying kinetic and thermodynamic competition between the constituent phases.

### 4.1 Mechanistic insights into sequential phase formation

**Fig. 5:**
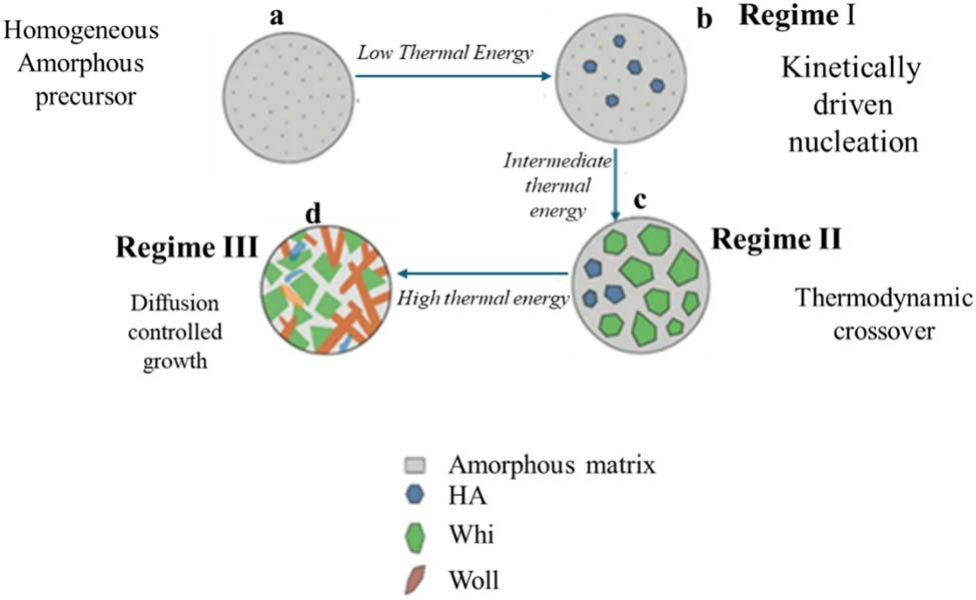
Mechanistic schematic of the sequential phase evolution in spray-pyrolyzed AWGCs. This diagram illustrates the predictable, three-stage crystallization pathway enabled by the exceptional homogeneity of the amorphous precursor. Each stage is governed by overcoming a distinct energetic barrier with increasing thermal input. (a) The process begins with a chemically uniform, amorphous precursor powder. (b) In regime I, low thermal energy (700–800 °C) overcomes the lowest activation barrier, resulting in the kinetically driven nucleation of the simplest phase, hydroxylapatite (HA). (c) In regime II, intermediate thermal energy (800–1000 °C) provides sufficient energy to trigger a thermodynamic crossover, leading to the pronounced crystallization of the more stable whitlockite (Whi) phase, which consumes a significant portion of the amorphous matrix. (d) In regime III, high thermal energy (>1000 °C) finally activates the complex, long-range mass transport required for the diffusion-controlled growth of the stable, high-temperature wollastonite (Wol) phase, which forms an interlocking network of reinforcing laths.

The exceptional homogeneity of the SP precursor is the key enabler for the predictable, sequential crystallization pathway observed in this study. By providing a structurally and chemically uniform amorphous matrix (Fig. 5a), our synthesis approach eliminates the stochastic nucleation events that typically confound phase evolution in conventionally prepared glass-ceramics. This enables a crystallization pathway governed not by random defects, but by the fundamental energetic landscapes of the competing crystalline phases.^14^

The thermodynamic crossover to the whitlockite-dominant regime between 800 °C and 1000 °C is clearly visualized in the XRD data (Fig. 1). The rapid consumption of the amorphous phase corresponds directly to the sharp increase in intensity and definition of the primary whitlockite peaks, particularly the (300) reflection. This suggests that the energy barrier for whitlockite nucleation is overcome in this temperature range, leading to its pronounced crystallization as predicted by our quantitative phase map (Fig. 2). The sequential emergence of HA, Whi, and Wol, as mapped in Fig. 2 and illustrated mechanistically in Fig. 5, can therefore be interpreted as a direct consequence of surmounting successive activation energy barriers with increasing thermal input.

At low thermal energies (regime I, 700–800 °C), the system is kinetically constrained. The initial crystallization is thus dominated by the phase with the lowest nucleation barrier: HA. As depicted in Fig. 5b, HA nucleation requires only local rearrangement of Ca^2+^ and PO_4_^3−^ ions, a process less energetically demanding than the long-range diffusion required for silicate polymerization.^15,16^ This kinetic preference allows HA to form as the primary crystalline phase, even if other phases may be more thermodynamically stable.

Upon reaching intermediate thermal energies (regime II, 900–1000 °C), the system gains sufficient energy to overcome a higher activation barrier, triggering a thermodynamic crossover. Here, the Mg^2+^ content of the precursor plays a critical role, destabilizing the HA lattice while promoting the formation of the more thermodynamically favorable Whi structure.^9,17^ The result is a pronounced crystallization of Whi, which rapidly consumes the residual amorphous phase and becomes a co-dominant component alongside HA Fig. 5c. This regime represents a thermodynamic equilibrium distinct from the kinetically limited state at lower temperatures.

The final transformation occurs at high thermal energies (regime III, >1000 °C), where the activation energy for significant, long-range mass transport is finally met. This activates the diffusion-controlled growth of Wol, an inosilicate whose formation necessitates the complex reorganization and polymerization of SiO_4_ tetrahedra into silicate chains.^18^ This high-barrier process, illustrated in Fig. 5d, yields an interlocking network of wollastonite laths, which confers mechanical reinforcement to the final ceramic. The growth of this stable high-temperature phase occurs at the expense of the pre-existing HA and Whi phases, demonstrating a dynamic solid-state reaction that drives the system toward its final, high-temperature equilibrium state. This well-defined, three-stage evolution from a homogeneous precursor confirms that phase selection in this system is a predictable function of overcoming discrete, mechanistically distinct energetic hurdles.^19,20^

### 4.2 Phase evolution–microstructure–densification

Crystallographic evolution is the direct cause of the observed microstructural densification. The peak density of 2.81 g cm^−3^ at 1000 °C corresponds precisely to the point of maximum crystallization before the onset of aggressive Wol growth. This indicates that crystallization-driven densification, where the formation of ordered crystal lattices eliminates free volume from the amorphous matrix, is the primary mechanism for pore removal. However, the slight density decreases at 1100 °C, despite the formation of more Wol, is a classic example of late-stage sintering phenomena. At this high temperature, grain boundary migration becomes exceptionally rapid, leading to aberrant grain growth that can isolate and entrap residual pores within the grains (Fig. 3c).^21,22^ This prevents the material from reaching its theoretical maximum density and highlights a critical trade-off: maximizing the mechanically reinforcing Wol phase comes at the minor cost of peak densification.^17,23^

### 4.3 A predictive tool for rational bio-ceramic design

This study's primary contribution is the translation of this fundamental understanding into a practical engineering tool. The phase-temperature map (Fig. 2) serves as a predictive guide for fabricating AWGCs with tailored functionalities. For instance, an engineer can now rationally select a processing protocol to achieve a specific clinical goal, as outlined in the design matrix (Table 1). This predictive capability represents a significant advancement over empirical approaches.

**Table 1:** A predictive processing-property map for spray-pyrolyzed AWGCs provides a comprehensive synthesis of the study's findings, correlating the prescribed processing temperature with (i) the experimentally determined quantitative phase composition, (ii) the observed microstructure and bulk density, and (iii) the measured *in vitro* biological response. This integrated data is then used to define the material's functional profile and suggest targeted biomedical applications, serving as a rational design guide. Biological response data (cell viability) is from our previous investigation^13^

Prescribed processing	Phase composition (wt%)	Observed microstructure & density	Measured biological response*	Resulting functional profile
800 °C (regime I/II boundary)	HA (∼38%), Whi (∼1%), Am (∼50%)	Nanocrystalline, porous surface with initial particle necking. Low density (∼2.35 g cm^−3^)	High biocompatibility (cell viability >170%)	Bioactive & resorbable. Ideal for a bioactive coating on an implant where rapid surface integration is needed without requiring structural integrity
900 °C (regime II)	Whi (∼28%), HA (∼35%), Am (∼25%)	Crystalline, fine-grained, moderate porosity. Increased density (∼2.62 g cm^−3^)	High biocompatibility (cell viability >250%)	Balanced bioactivity & resorption. Suitable for a resorbable bone graft or filler where both hydroxylapatite and the more resorbable whitlockite phase are beneficial
1000 °C (regime II/III boundary)	Whi (∼25%), HA (∼20%), Wol (∼10%), Am (∼15%)	Highly dense, consolidated grains with minimal porosity. Peak Density (∼2.81 g cm^−3^)	Excellent biocompatibility (cell viability >260%)	Optimal density & stability. Best suited for a component where density and stability are paramount, while maintaining excellent bioactivity. A potential non-load-bearing implant
≥1100 °C (regime III)	Wol (∼22%), Whi (∼15%), HA (∼18%), Am (∼10%)	Dense, coarse-grained with reinforcing laths and some entrapped porosity. Slightly reduced density (∼2.70 g cm^−3^)	Peak biocompatibility (cell viability >300%)	Highest stability & bioactivity. The presence of ∼22 wt% wollastonite suggests enhanced mechanical stability. Ideal starting point for developing load-bearing applications

## 5. Conclusion

In summary, this work establishes a clear and quantitative framework for the rational design of multiphase AWGcs. By harnessing the exceptional homogeneity of spray-pyrolyzed precursors, we have disentangled the kinetic and thermodynamic factors that drive crystallization, resulting in predictable and repeatable material outcomes. Our findings reveal three distinct thermal regimes, each marked by the sequential dominance of HA, Whi, and Wol. The resulting quantitative phase-temperature map transforms the fabrication of AWGCs from an empirical process into a science-based, predictive approach.

This map provides engineers with a powerful tool for tailoring phase composition, enabling the production of bio-ceramics with precisely controlled biological and mechanical properties for advanced medical applications. Looking ahead, future work will use this predictive framework to create AWGCs with targeted phase compositions, subjecting them to comprehensive mechanical evaluations such as nanoindentation and fracture toughness testing. Ultimately, this will allow us to fully validate the processing-structure–property-performance relationship and advance the engineering of next-generation bio-ceramics.

## Author contributions

Conceptualization, methodology, data analysis, and writing original draft: Andualem Belachew Workie; review & editing: Mannie Belay Taye.

## Conflicts of interest

The authors declare that they have no known competing financial interests or personal relationships that could have appeared to influence the work reported in this paper.

## Supplementary Material

RA-016-D5RA08885B-s001

## Data Availability

All data supporting the findings of this study are included in the manuscript; additional raw data can be provided upon reasonable request. Supplementary information (SI): supporting characterization data for the AWGC precursors and sintered glass-ceramics. Fig. S1: particle size distribution histogram of the initial powder. Fig. S2: representative EDS spectrum and quantitative elemental analysis confirming chemical homogeneity. Table S1: detailed phase composition, crystallite size, and bulk density for samples sintered between 700 °C and 1100 °C, including Rietveld refinement quality parameters *χ*^2^. Table S2: comparison of theoretical and experimental elemental concentrations (Mol %). See DOI: https://doi.org/10.1039/d5ra08885b.
