# Supplementary material for: Sequential crystallization pathways in apatite–wollastonite glass-ceramics via spray pyrolysis
Source: RSC Adv. 2026 Mar 18;16(17):15242–8. doi: 10.1039/d5ra08885b (PMC12997414; doi:10.1039/d5ra08885b)
Supplement: RA-016-D5RA08885B-s001 [file RA-016-D5RA08885B-s001.pdf]

Supplementary Information (SI) for RSC Advances.
This journal is © The Royal Society of Chemistry 2026

Supplementary Information

Sequential Crystallization Pathways in Apatite–Wollastonite Glass-Ceramics via Spray Pyrolysis Manuscript ID: RA-ART-11-2025-008885

Figures

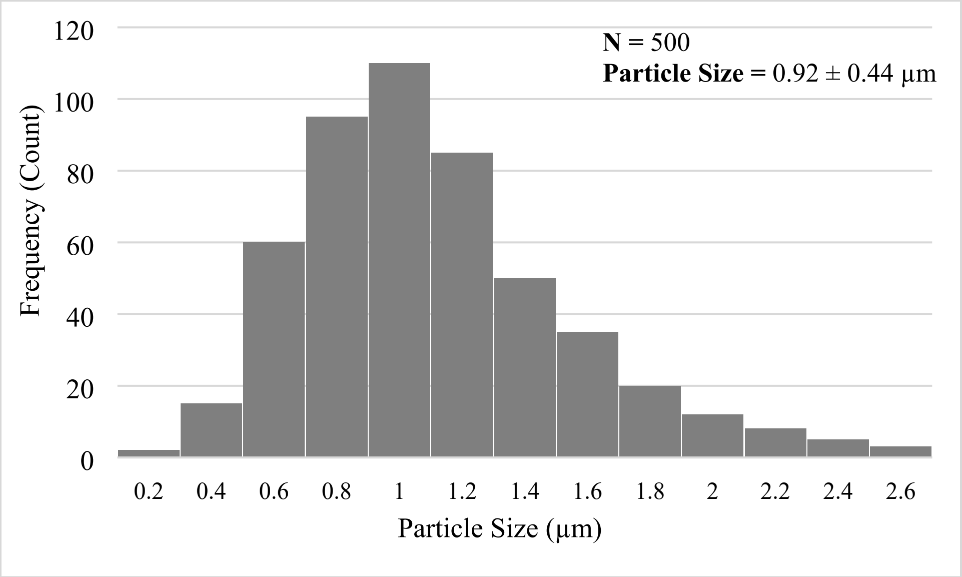

Figure S1: The particle size distribution histogram for the initial powder.

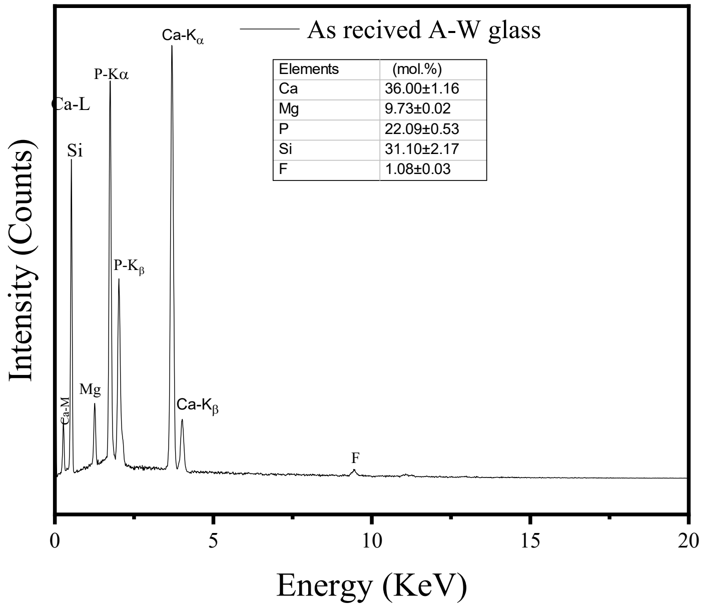

Figure S2. Representative Energy Dispersive X-ray Spectroscopy (EDS) spectrum of the as-synthesized apatite-wollastonite glass-ceramic (AWGC) precursor powder. The spectrum confirms the presence of all constituent elements (Ca, Si, P, Mg, and F). The quantitative analysis (inset. table, Mol %) demonstrates that the elemental composition is in excellent agreement with the theoretical stoichiometry, confirming the chemical homogeneity achieved via the spray pyrolysis method. This data supports the compositional analysis presented in Table S1.

Tables

Table S1. Detailed structural and phase parameters of sintered AWGCs.

Parameter	700 °C	800 °C	900 °C	1000 °C	1100 °C
**Phase Composition (wt.%)**					
Amorphous Phase	54.40	59.60	36.00	30.80	22.60
Hydroxyapatite (HA)	37.31	6.73	22.40	20.18	12.38
Whitlockite (Whi)	8.29	23.57	32.00	34.60	43.34
Wollastonite (Wol)	4.14	10.10	9.60	14.42	21.67
**Crystallite Size (nm)**					
Hydroxyapatite (D_v)	25.41	28.76	32.15	35.42	36.79
Bulk Density (g/cm³)	2.01	2.35	2.62	2.81	2.70
**Refinement Quality**					
Goodness of Fit (χ^2)	1.15	1.12	1.09	1.08	1.10

Table S2. EDS Elemental Analysis of AWGC Precursors.

Element	Theoretical (Mol %)	Experimental (Mol %)
**Calcium (Ca)**	36.23	36.00 ± 1.16
**Silicon (Si)**	35.30	31.10 ± 2.17
**Phosphorus (P)**	13.74	22.09 ± 0.53
**Magnesium (Mg)**	13.70	9.73 ± 0.02
**Fluorine (F)**	1.03	1.08 ± 0.03
